# A Quantum Cascade Laser-Based Optical Sensor for Continuous Monitoring of Environmental Methane in Dunkirk (France)

**DOI:** 10.3390/s16020224

**Published:** 2016-02-08

**Authors:** Rabih Maamary, Xiaojuan Cui, Eric Fertein, Patrick Augustin, Marc Fourmentin, Dorothée Dewaele, Fabrice Cazier, Laurence Guinet, Weidong Chen

**Affiliations:** 1 Laboratoire de Physicochimie de l’Atmosphère, Université du Littoral Côte d’Opale, Dunkerque 59140, France; rabih.maamary.physique@gmail.com (R.M.); fertein@univ-littoral.fr (E.F.); augustin@univ-littoral.fr (P.A.); fourment@univ-littoral.fr (M.F.); 2 Anhui Institute of Optics and Fine Mechanics, Chinese Academy of Sciences, Hefei 230031, China; 3 Centre Commun de Mesures, Université du Littoral Côte d’Opale, Dunkerque 59140, France; Dorothee.Dewaele@univ-littoral.fr (D.D.); cazier@univ-littoral.fr (F.C.); 4 ONERA, DOTA, BP 80100, Palaiseau cedex 91123, France; Laurence.Guinet@univ-littoral.fr

**Keywords:** QCL-based sensor, environmental methane, campaign measurement

## Abstract

A room-temperature continuous-wave (CW) quantum cascade laser (QCL)-based methane (CH_4_) sensor operating in the mid-infrared near 8 μm was developed for continuous measurement of CH_4_ concentrations in ambient air. The well-isolated absorption line (7F_2,4_ ← 8F_1,2_) of the ν_4_ fundamental band of CH_4_ located at 1255.0004 cm^−1^ was used for optical measurement of CH_4_ concentration by direct absorption in a White-type multipass cell with an effective path-length of 175 m. A 1σ (SNR = 1) detection limit of 33.3 ppb in 218 s was achieved with a measurement precision of 1.13%. The developed sensor was deployed in a campaign of measurements of time series CH_4_ concentration on a site near a suburban traffic road in Dunkirk (France) from 9 to 22 January 2013. An episode of high CH_4_ concentration of up to ~3 ppm has been observed and analyzed with the help of meteorological parameters combined with back trajectory calculation using the Hybrid Single Particle Lagrangian Integrated Trajectory (HYSPLIT) model of NOAA.

## 1. Introduction

Methane (CH_4_), as the second most important greenhouse gas after carbon dioxide (CO_2_), contributes to the increase in global warming with a lifetime of about 12 years [1,2]. Its concentration in the atmosphere has increased since the pre-industrial time from 700 ppb (parts per billion) to 1810 ppb [3]. Methane is produced naturally by anaerobic decomposition of organic materials as well as emissions from anthropogenic sources like ruminants, rice agriculture, biomass burning, fossil fuels and landfills [4,5]. CH_4_ is also the main constituent of natural (82%) and liquefied natural gas (95%) used nowadays in thermoelectric plants all over the world. Methane oxidation in the tropospheric and stratospheric layers is a key-reaction that affects the atmospheric concentration of the hydroxyl (OH) radicals and hence the oxidative capacity of the atmosphere. At the regional level, a liquefied natural gas (LNG) terminal located in Dunkirk (France) will be in operation in 2016. Risks of methane leakage from the storage tanks, from the pipelines during the transportation, and from burning methane in excess in flares when performing new filling cycles in the reservoirs are not negligible. Reliable, fast and high-precision continuous monitoring of CH_4_ concentration is therefore highly required for local industrial security management in this LNG installation context, but also for monitoring of surrounding environmental changes to well understand the environmental impact of additional CH_4_ emissions from natural gas production, from distribution and end use. Moreover, continuous measurements may give useful new insights into the global CH_4_ budget on a regional scale.

Recommended by World Meteorological Organization [6], atmospheric methane concentration is typically measured by gas chromatography-flame ionization detection (GC/FID). CH_4_ in ambient air is separated using chromatographic scheme prior to flame ionization detection. The measurement is quasi-continuous (~15 min per datum) due to the sample preparation by the chromatographic separation processes.

Compared to the conventional GC/FID method, optical methods based on spectroscopic techniques [7,8] offer the capability of fast and continuous monitoring of environmental gaseous mixture without sample preparation, which makes the optical sensing immune to chemical interference/contamination due to sample preparation and conversion (hence high specificity). Nowadays, optical gas sensors based on newly emerging infrared lasers allows one to probe strong fundamental rotational-vibrational transitions of the target molecules and thus to achieve very high measurement sensitivity (detection limit down to ppb and parts per trillion (ppt) levels). These field deployable optical gas sensors with a reduction in size and cost have become ease-to-use and can be operated by non-technical personnel [9]. Various spectroscopic techniques have been developed in order to achieve high detection sensitivity for CH_4_ detection, such as photoacoustic spectroscopy (PAS) or quartz-enhanced photoacoustic spectroscopy (QEPAS) [10,11,12], open-path differential optical path absorption spectroscopy (DOAS) [13] and long-path absorption spectroscopy using a multipass cell [14,15,16] or a high-finesse optical cavity as in cavity ring-down spectroscopy (CRDS) [17] and off-axis integrated cavity output spectroscopy (OA-ICOS) [18].

In this paper, we report on the development and deployment of a continuous-wave (CW) quantum cascade laser (QCL)-based CH_4_ sensor operating near 8 µm. The objective of the present work is to develop an optical sensor based on direct multipass absorption spectroscopy capable of performing *in situ* continuous measurement of CH_4_ absolute concentration in the environment, which could provide a local background reference of CH_4_ emission level prior to the installation of a new LNG terminal in Dunkirk in 2016. The choice of direct absorption spectroscopic scheme makes the sensor platform self-calibrated (unlike PAS or QEPAS requiring additional calibration). The use of a multipass-based long-path absorption approach operating at optimal gas pressure (to enhance measurement selectivity) permits highly sensitive absolute concentration measurement (unlike DOAS that measures integrated concentration over an open path) while keeping the sensor system simple and attractive for applications in harsh environment (advantageous over cavity enhanced setups where protecting the high-reflectivity cavity mirrors against contamination by dust is always a serious issue to be addressed)*.*

## 2. Experimental Details

### 2.1. Experimental Setup

**Figure 1:**
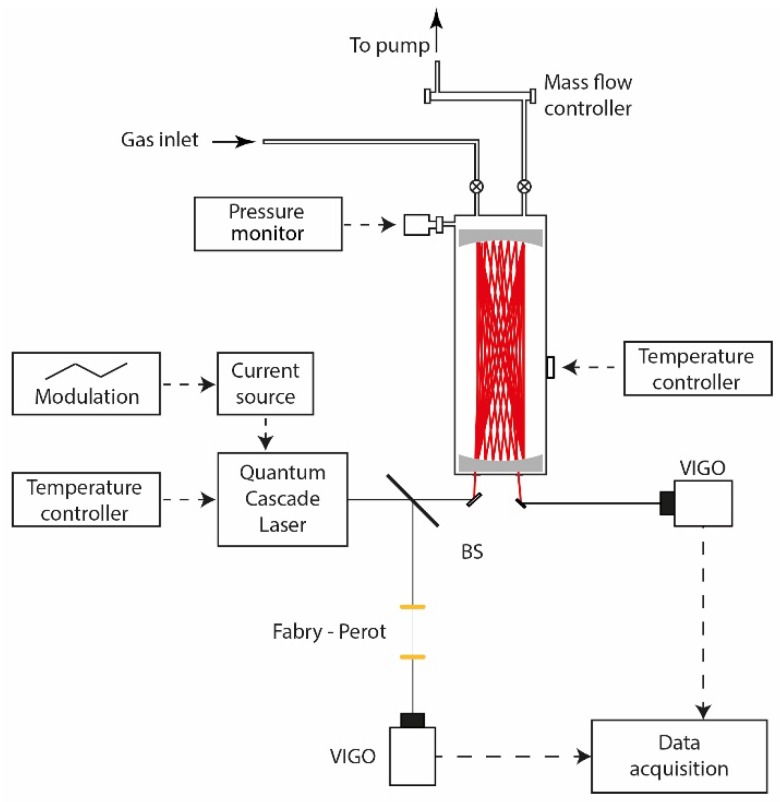
Experimental setup used for ambient CH_4_ monitoring (BS: beam splitter).

The experimental setup developed in the present work is depicted in Figure 1. The used distributed feedback quantum cascade laser (DFB-QCL, DQ7- M776H, Maxion Technologies, Inc., Jessup, MD, USA) is tunable from 1252.1 to 1257.1 cm^−1^ (at ~8 µm) by adjustment of the laser temperature and current. The laser operates at room temperature in continuous mode with an output power of 35 mW when powered at 8.895 V and 333 mA. Its spectral coverage allows for optical sensing of multi-species: water vapor (H_2_O), methane (CH_4_), nitrous acid (HONO) and nitrous oxide (N_2_O). Fast laser frequency tuning at the kHz-scan-rate per spectrum can be achieved using a triangular current modulation, which is favorable for applications where high temporal resolution is required, as in flux measurements.

As shown in Figure 1, a beam splitter (10/90) was used to divide the incident QCL beam into two parts. The 10% was sent to a Fabry-Perot etalon for frequency metrology of the DFB-QCL. The main part (90%) was directed into a White-type multipass cell to enhance the measurement sensitivity by long optical path-length absorption spectroscopy.

The multipass cell (107-V Infrared Analysis, Inc., Anaheim, CA, USA) is made of borosilicate glass with a volume of about 25 L. The effective optical path-length *L*_eff_ can be adjusted from 1.6 to 175 m by changing the orientation of the multipass cell mirrors. The emerging laser beam was focused onto a thermoelectrically cooled infrared photovoltaic detector (VIGO PVI 4-TE-10.6) with a detectivity of 2 × 10^9^ cm·Hz^1/2^/W and a responsivity of 0.7 A/W. A high-speed digital oscilloscope (LeCroy WaveSurfer 104 MXs 1 GHz Oscilloscope 5 GS/s, Teledyne Technologies Inc., Thousand Oaks, CA, USA), controlled with a LabVIEW program, was used for spectral data acquisition. Post processing of the recorded data was carried out to retrieve CH_4_ concentration based on the Beer-Lambert law.

**Figure 2:**
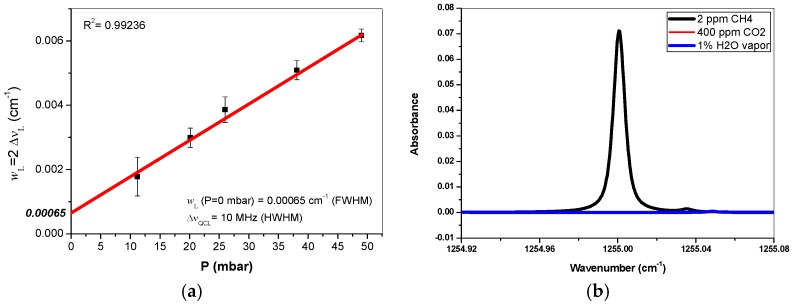
(**a**) Plot of CH_4_ absorption line width *vs.* pressure for determination of the DFB-QCL emission line width; (**b**) Simulation of 2 ppm CH_4_ absorption spectrum around 1225 cm^−1^ at 40 mbar in a multipass cell with 175 m effective optical path-length.

The line width of the QCL emission, playing an important role in spectral profile fit for gas concentration retrieval, was experimentally investigated. Methane samples (3000 ± 60 ppm) were injected into the multipass absorption cell at different pressures, and the corresponding absorption spectra were recorded. The spectra were fitted to a Voigt line profile in order to determine the Lorentzian line width *w*_L_ (Full Width at Half Maximum-FWHM). The plot of the Lorentzian line width as a function of the pressure, as shown in Figure 2a, allows us to deduce the laser emission line width of ~10 MHz (at the pressure equal to zero) [19].

Outdoor environmental air was sampled from the campaign site and drawn to the inlet of the multipass cell via a Teflon tube. The outlet of the cell was connected to a primary pumping system to maintain a regular sampling gas flow at a constant rate of 41.6 L/min, resulting in a gas residence time of about 36 s. Temperature and pressure of the gas sample inside the multipass cell were typically set at 30 °C and 42 mbar (optimized as a comprise between spectral selectivity and detection sensitivity), respectively. The temperature was controlled by using a heating jacket with a silicone-based heating blanket which is controlled by thermocouple to heat the cell chamber uniformly. A digital temperature control unit (Infrared Analysis, Inc.) maintains temperature with a precision of ±0.01 °C.

### 2.2. Selection of Methane Absorption Line for Sensitive and Selective Monitoring

Selection of suitable absorption lines is crucial for spectroscopic monitoring of trace gas. The absorption line(s) should be as strong as possible and well isolated as well in order to avoid any spectral interference from itself or from other abundant atmospheric species (for instance CO_2_, H_2_O vapor) present in the atmosphere. Figure 2b shows a simulated spectrum of the selected CH_4_ absorption line (the transition 7F_2,4_ ← 8F_1,2_ of the ν_4_ fundamental band, centered at 1255.0004 cm^−1^ with a line intensity of 2.07 × 10^−20^ cm·molecule^−1^) [20]. The simulation was made with 2 ppm CH_4_ in an absorption cell with an effective optical path-length of *L*_eff_ = 175 m at 40 mbar. Potential interferences from ambient CO_2_ (at 400 ppm) and 1% H_2_O vapor are taken into account in the simulation. Though the absorption intensity of the selected CH_4_ line is not the strongest, it is strong enough for detection of atmospheric CH_4_ at its natural concentration level (~1.7 ppm). As can be seen, this line is free of interference from the absorptions of CO_2_ and H_2_O. It is a good tradeoff between the required sensitivity and specificity. In the present work, this methane absorption line located at 1255.0004 cm^−1^ was used to monitor environmental CH_4_ by direct absorption spectroscopy in a *L*_eff_ = 175 m long multipass cell.

### 2.3. Frequency Metrology and Absorption Spectrum Retrieval

**Figure 3:**
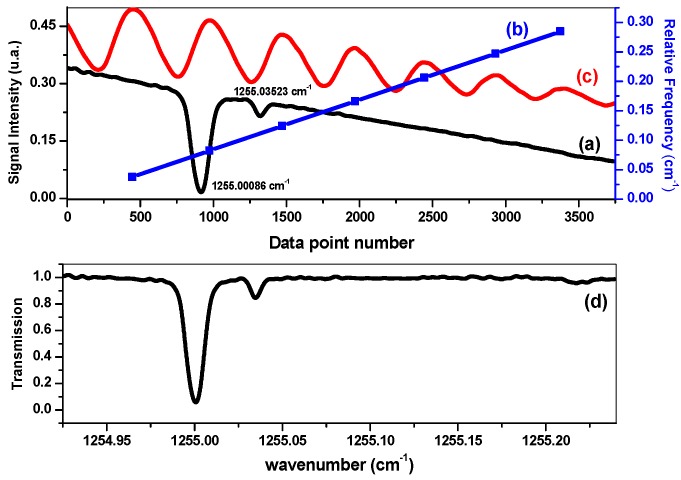
Absorption signal of 3000 ppm CH_4_ at 4.62 mbar (**a**); converted into an absorption spectrum (**d**) using the relative frequency calibration curve (**b**) which allows one to convert “data point number” into “wavenumber” by means of the interference fringes resulting from a Fabry-Perot interferometer with a FSR of ~0.033 cm^−1^ (**c**).

The CH_4_ absorption signal was recorded as a function of the acquisition data number ((a) in Figure 3) during the scan of the QCL frequency by laser current tuning. 

In order to convert this signal into an absorption spectrum ((d) in Figure 3), interference fringes from a Fabry-Perot interferometer were recorded ((c) in Figure 3) and used to determine the relationship between the data number and the related frequency ((b) in Figure 3). The absolute frequency was determined using the CH_4_ absorption positions given in the HITRAN database [20]. The interferometer is built with two high transmission ZnSe plates (~10% reflectivity at 8 µm) separated by 15 cm, resulting in a free spectral range (FSR) of ~0.033 cm^−1^.

### 2.4. Concentration Retrieval

Retrieval of trace gas concentration from the measured absorption spectra is based on the Beer-Lambert law [21]:

(1) $$I(\nu )=I_{0}\exp(-CL\sigma (\nu ))=I_{0}\exp(-A(\nu ))$$

and:

(2) $$A(\nu )=ln\left(\frac{I_{0}}{I(\nu )}\right)=LC\sigma (\nu )$$

where I_0_(ν) and I(ν) are the transmitted laser intensities without and with target gas molecules in the absorption cell, respectively. *L* is the effective optical path-length in (cm), *C* is the gas-molecule concentration in (molecule/cm^3^), *A(*ν*)* is the absorbance, and σ*(*ν*)* in (cm^2^/molecule) is the frequency-dependent absorption cross section of the target gas molecule. 

Integral absorbance *A_I_* of the absorbance *A(*ν*)*, in (cm^−1^), over all the scanned spectral range can be written as:

(3) $$A_{I}=\int A(\nu )d\nu =\int LC\sigma (\nu )d\nu$$

where ∫σ(ν)dν=S, in (cm/molecule), is the absorption line intensity. The concentration of the target gas molecule χ_[ppm]_ in [ppm] (parts per million) can be expressed as:

(4) $$\chi_{\left[ppm\right]}=\frac{C}{N(T,P)}\times 10^{6}=\frac{{Α}_{I}}{SLN(T,P)}\times 10^{6}$$

with *N* the total molecules number per unit volume (molecules/cm^3^) at pressure *P* and temperature *T*:

(5) $$N(T,P)=N_{L}\frac{P}{P_{0}}\frac{T_{0}}{T}$$

where *N_L_* is the Loschmidt constant (2.68678 × 10^19^ molecules/cm^3^), *P_0_* and *T_0_* are the pressure and the temperature at standard conditions (1013.25 mbar and 273.15 K) respectively.

### 2.5. Measurement Accuracy

As shown in Equation (4), the accuracy of the concentration determination strongly depends on the accuracy of the optical absorption length *L*, the absorption line intensity *S* and the quality of the spectra recorded (*i.e*., signal-to-noise ratio, SNR). 

**Figure 4:**
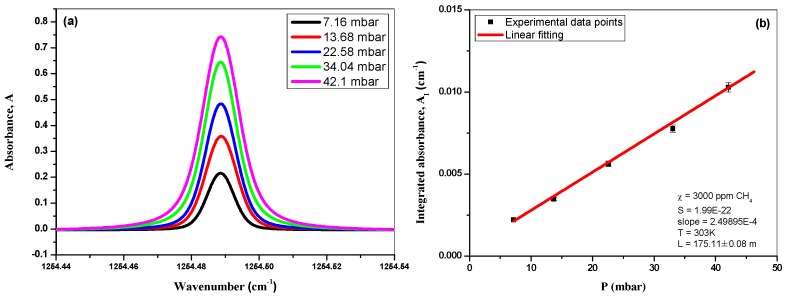
(**a**) 3000 ppm methane absorption spectra recorded at different pressures; (**b**) Plot of the integrated absorbance *vs.* pressure *P*, associated with a linear fit (corresponding to the *A*_I_/*P* ratio).

In the present work, absorption spectra of 3000 ppm CH_4_ located at 1254.4887 cm^−1^ (6F_3_ ← 7F_1_) were recorded at different pressures (Figure 4a) for accurate determination of the effective absorption path-length *L*_eff_:

(6) $$L_{eff}(cm)=\frac{A_{I}}{P}\times \frac{P_{0}T}{\chi SN_{L}T_{0}}\times 10^{6}$$

Based on the results shown in Figure 4b, an effective path-length of *L*_eff_ = 175.11 ± 0.08 m was found. The corresponding uncertainty was estimated to be 28 ppb due to the uncertainty in *L*_eff_. The total uncertainty on the concentration value deduced from the spectroscopic measurement is about 5.1% which is directly related to the uncertainties in the Voigt profile fitting (Δ*A*_I_ < 1%) depending on the SNR of the spectra, in the line intensity reported in the HITRAN database (Δ*S* < 5%), in Δ*L*_eff_ (= 0.05%), in the values of pressure (Δ*P* = 0.2%) and temperature (Δ*T* = 0.03%).

### 2.6. Measurement Precision

**Figure 5:**
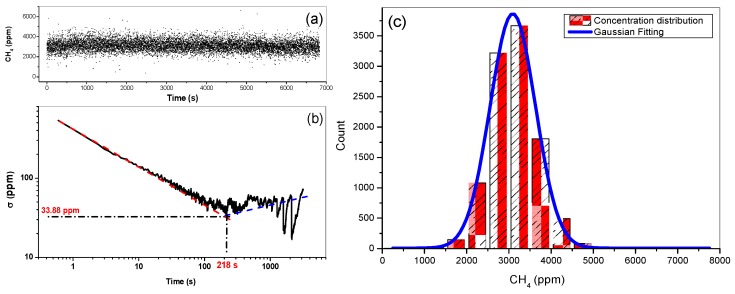
(**a**) Time series measurements of CH_4_ concentration; (**b**) Allan deviation plot; (**c**) Histogram showing the distribution of the methane concentration retrieved from 10,540 absorption spectra recorded with 0.65-s integration time at ambient temperature and at a pressure of 42 mbar.

Conventional data averaging techniques were used to reduce white noise in order to enhance the SNR in the recorded spectral data. Laser frequency was scanned over 0.2 cm^−1^ around 1254.5 cm^−1^ at a relatively high rate of 2.5 kHz. Allan variance analysis was performed [22,23,24,25,26] to determine the optimal averaging time. Time-series measurements of 3000 ppm CH_4_ absorption spectra were carried out for the Allan variance study. Based on 10,540 consecutive 0.65-s recorded CH_4_ spectra (Figure 5a), an Allan variance σ^2^ was calculated as a function of the averaging time [23] and the Allan deviation σ is plotted in Figure 5b. As can be observed in Figure 5b, the Allan deviation curve (black), represented in logarithmic scale, shows two different behaviors. At first, a linear decay part (red dashed line) indicates linear decrease in deviation with the averaged number of the data, *i.e.*, efficient white noise reduction and hence the improvement in the measurement precision. After that, the instabilities of the instrumental system nevertheless counterbalance the noise reduction given by average and the deviation increases, as shown in blue dashed line. 

The optimal data averaging time for the present QCL sensor was found to be about 218 s. Under this optimal averaging condition, the σ value, corresponding to the measurement precision, was about 33.88 ppm, leading to a relative uncertainty in the measurement precision of 1.13%. The distribution histogram of the measured concentrations is plotted in Figure 5c and fitted to a Gaussian distribution function (blue curve), the most frequent concentration of 3092.82 ± 6.31 ppm was deduced from the highest counting rate of 3600 times. Compared to the manufactured concentration of 3000 ± 60 ppm, the discrepancy between our measurement and the supplier’s specific value is about 3.0%, which is within the measurement uncertainty of 5.1% as discussed above in Section 2.5.

**Figure 6:**
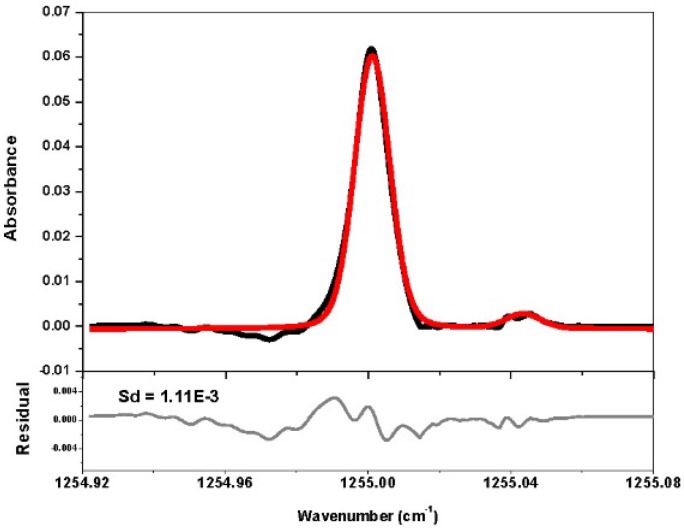
*Upper panel*: Absorption spectrum of CH_4_ (black curve) in ambient air (750 averaged spectra over 218 s) and Voigt profile fitting (red) to the experimental absorption line. *Lower panel*: Fit residual.

Figure 6 shows an experimental absorption spectrum of ambient CH_4_. With 750 spectra averages over 218 s, the concentration was retrieved to be (1870 ± 16) ppb with a 1σ detection limit of about 33.3 ppb.

## 3. Continuous Monitoring of CH_4_ at a Suburban Site in Dunkirk-France

### 3.1. Campaign Site Description

**Figure 7:**
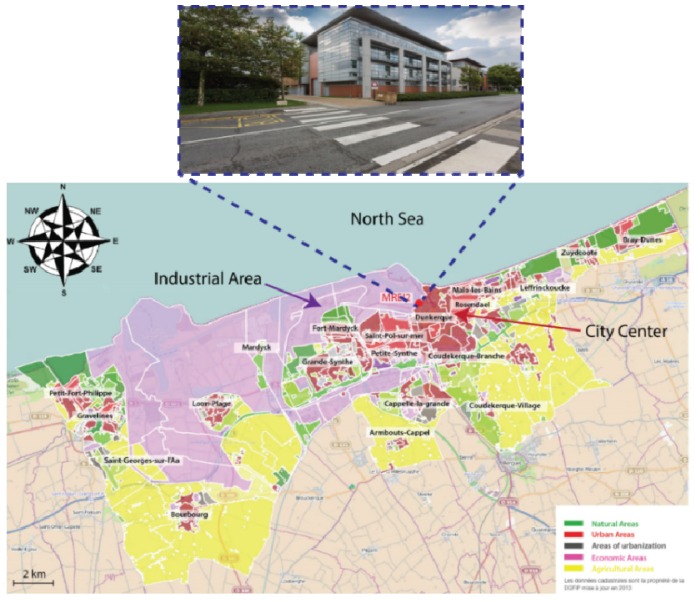
Campaign site.

The developed QCL-CH_4_ sensor was deployed for continuous monitoring of environmental CH_4_ during a field campaign of about 2 weeks (from 9 to 22 January 2013) on a suburban site in Dunkirk, France (Figure 7).

It is near a moderate-traffic road (latitude/longitude: 51.0358613/2.3654160), 3 km from the well-developed industrial area (situated at the western side of the sampling site), 2 km from the North Sea (at the north of the site) and about 500 m from the city center (south-south-east of the site). Ambient air was sampled using Teflon tubing, fixed at about 180 cm above the ground, connected to the QCL-CH_4_ sensor. Measurements of the environmental CH_4_ were performed using the developed QCL-sensor to probe the methane absorption line centered at 1255.0004 cm^−1^ by direct absorption over an effective optical absorption path-length of *L*_eff_ = 175 m in a multipass cell with a data acquisition rate of 0.65 s/spectrum.

### 3.2. Complementary Measurements

Basic meteorological parameters (such as temperature, wind direction, wind speed, water vapor mixing ratio, global solar radiation) were measured with a sampling resolution of 15 min throughout the measurement campaign. The used weather station (Davis Vantage Pro2, Montanay, France) was installed on the top of the laboratory building next to the sampling site at about 20 m above the air sampling tube in order to avoid any wind perturbation because of surrounding buildings. For the wind speeds lower than 4 m/s (the resolution limit of Davis Vantage Pro2), the wind direction and wind speed data measured at a rate of 20 Hz by a METEK ultrasonic anemometer (uSonic-3 Scientific USA-1, Elmshorn, Germany) were used. This ultrasonic anemometer collecting the turbulence parameters in the boundary layer was installed on a 15 m tower, located about 1.5 km north from the campaign site. The effect of the distance (~1.5 km) between two measurement instruments (METEK ultrasonic anemometer and Davis Vantage Pro2) has been investigated. Good correlation between the two instruments’ measurements were observed with *R*^2^ = 0.95 and 0.85 for the measurements of wind direction and wind speed, respectively, during the full campaign period.

Carbon monoxide (CO) and nitrogen oxides (NO, NO_x_) were measured with a CO analyzer (CO 12M-Environnement, SA, Poissy, France) with a 1σ detection limit of 25 ppb and a NO_x_ analyzer (AC 31M—Environnement SA, 1σ detection limit of 0.17 ppb)*.* Sulfur dioxide (SO_2_) was also measured with a 1 σ detection limit of 0.5 ppb using an AF 22M SO_2_ Analyzer (Environnement, SA) in order to determine whether the industrial area has an influence on the methane budget, as well as ozone (O_3_) measured by an O_3_ analyzer (41 M—Environnement, SA, with a 1σ detection limit of 0.5 ppb). All gas measurements were carried out with a sampling resolution of 15 min.

### 3.3. Results and Discussion

**Figure 8:**
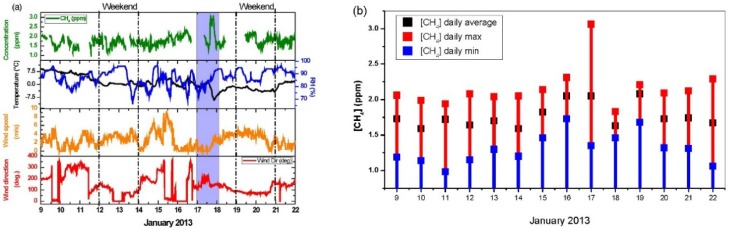
(**a**) Time-series methane concentrations variation sampled at 15-min intervals; (**b**) CH_4_ daily average concentration (black dots), daily observed maximum (red) and minimum (blue) concentrations.

Figure 8a presents time-series measurements of CH_4_ concentration (green) accompanied with the measured temperature (black), humidity (blue), wind speed (orange) and direction (red). All data are averaged over 15 min. In Figure 8b, we present the daily averaged methane concentration associated with the maximum (red) and minimum (blue) values. 

**Figure 9:**
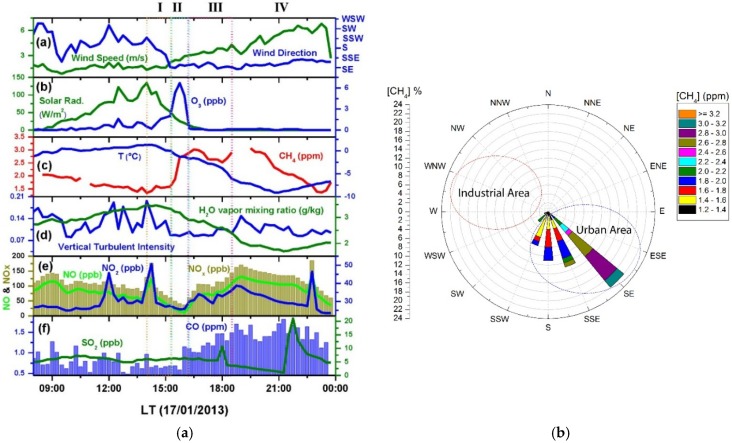
Time series (in local time, LT) measurements (**a**) and pollution-concentration wind rose plot (**b**) showing the dependence of CH_4_ concentration on the wind direction on 17 January 2013.

The averaged CH_4_ concentration (black) was found to be (1.77 ± 0.20) ppm during the 14 days’ campaign. High daily averaged CH_4_ concentration bigger than 2.08 ppm was observed on 16, 17, 19 and 22 January. The highest CH_4_ concentration of up to ~3 ppm was observed starting from ~15:00 (Paris winter local time) on 17 January along with a temperature decreasing down to −9 °C. This episode of high temporal variability of CH_4_ has been examined with the help of meteorological parameters as well as the time-series concentration variations of NO_x_, NO_2_, NO, CO, SO_2_ and H_2_O vapor, as plotted in Figure 9a.

Starting from about 14:00 to 15:00 (Figure 9a, Period I), there was a change of wind direction from south-south-west (SSW) to south-east (SE), as shown in Figure 9a blue curve, leading to a change of air mass accompanied by a drop of temperature ((c) blue curve) and water vapor mixing ratio (from 3.5 down to 2 g/kg, Figure 9d green curve), an increase of CO ((f) blue column).

The observed increase in CH_4_ concentrations may originate from the urban area (SE) brought by this new air mass (see the pollution wind rose in Figure 9b). During period II after the change of air mass in period I, the low wind speed (<3 m/s, (a) green line) and low temperature (<−1 °C, (c) blue line) may promote accumulation of pollutants on the measurement site. This accumulation effect was confirmed by the low vertical turbulent intensity during this period ((d) blue line), deduced from the METEK ultrasonic anemometer; this implies a reduction of turbulent mixing thereby inhibiting dilution, leading to the increase in NO_x_, NO, CO concentrations and high CH_4_ concentration during all the period III.

From about 19:00, a progressive change of the wind direction from south-east (SE) to south-south-east (SSE), associated with an increase in wind speed and in vertical turbulent intensity, may lead to an increase of pollutants dispersion which contributes to the observed decrease in concentrations of CH_4_ as well as CO and NO_x_. Note that there was no significant wind blowing from the industrial areas located at the west side of the sampling site, and hence no industrial contribution to the measurement results was likely during this observation period. This analysis is confirmed by the CH_4_-concentration wind rose shown in Figure 9b.

**Figure 10:**
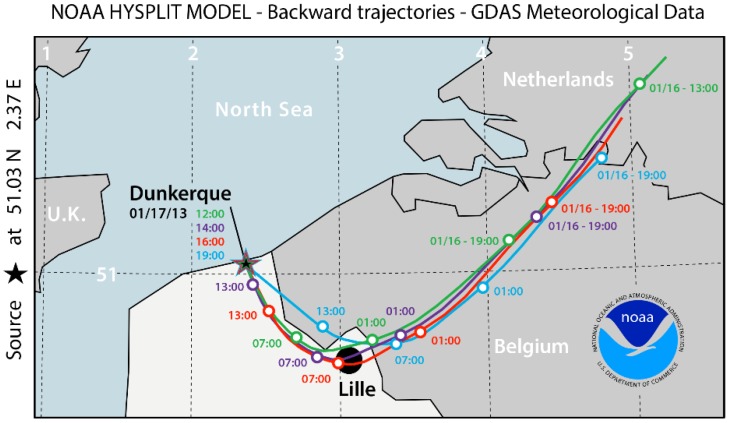
Air mass back trajectories (NOAA HYSPLIT) for the observed episode during the period of 12:00–19:00 on 17 January 2013.

In order to better understand the highest methane concentration (of the whole field campaign period) measured during this day, back trajectories of the air mass on 17 January were calculated (Figure 10) using the Hybrid Single-Particle Lagrangian Integrated Trajectory (HYSPLIT) model of National Oceanic and Atmospheric Administration (NOAA) [27].

These air-mass back trajectories show the contribution of transport paths to the observed high CH_4_ concentration peak by adding the pollutants imported by the air mass passing through Lille (arriving in Dunkirk during the period of 14:00–18:00), the fourth largest city in France, with a population of more than 1 million inhabitants.

**Figure 11:**
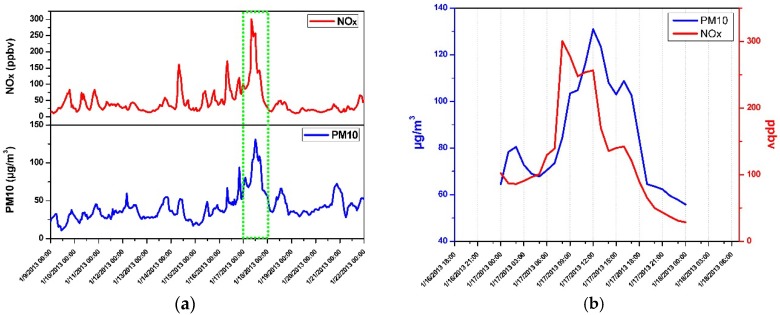
(**a**) Lille air quality monitoring data of NOx and PM10 (“Source Atmo Nord–Pas de Calais”) during the campaign period of 19–22 January 2013, recorded by the observation station of the ATMO Nord–Pas-de-Calais [28] in Lille Five. A high pollution episode has been also recorded on 17 January 2013, the same day as the biggest CH_4_ concentration observed in Dunkirk; (**b**) detailed concentration evolution of NOx and PM10 on this very day.

In order to further confirm the arrival of the pollutant from Lille, officially available data of NOx (NO and NO_2_) and PM10 (CH_4_ is not monitored for the regional air quality monitoring) measured during our campaign period (9–22 January 2013) by a network monitoring site in Lille Five (Latitude/Longitude: 50°37′41′′/3°5′26′′) have been explored in comparison with our measurement data. As shown in Figure 11a, a highest pollution episode (highest levels of NOx and PM10) has been also observed on 17 January 2013, the same day as the biggest CH_4_ concentration observed in Dunkirk (Figure 8).

**Figure 12:**
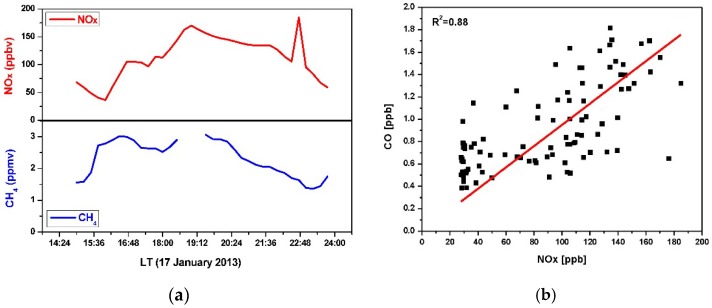
Correlation of NOx *vs.* CH_4_ (**a**) and NOx *vs.* CO (**b**) during the period of the observed episode on 17 January 2013.

It is well known, combustion emission is the primary sources of both CH_4_ and NOx [4,29,30]. Figure 12a shows such a positive correlation between NOx and CH_4_, observed during the highest CH_4_ concentration episode on this very day. It is worth noting that the temperature dropped from ~0 °C to −9 °C within 6 h from 15:00 to 21:00, the period in which the CH_4_ concentration peak has been observed.

Figure 11b details the concentration evolution of NOx and PM10 in Lille on this very day, which confirms the results of the air mass back trajectories shown in Figure 10: the air mass trajectory (red) passing by Lille near 7:00 (corresponding to a significant increase of NOx level in air) arrive at Dunkirk at about 16:00 where the CH_4_ concentration getting started raising. Furthermore, good correlation between NO_x_ and CO has been observed (Figure 12b), which can be an indicator of a common source of emission of these pollutants.

**Figure 13:**
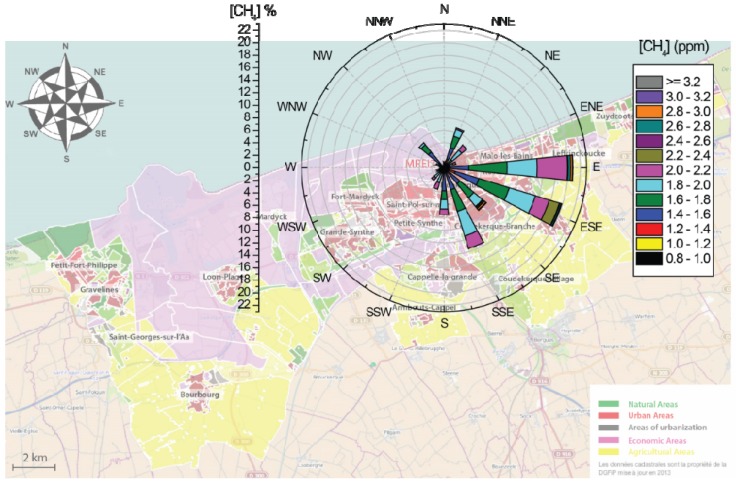
Frequency distribution rose of CH_4_ concentrations depending on the blowing wind sectors from 9 to 22 of January 2013 in Dunkirk.

The CH_4_ concentrations in relation to wind sector during all the measurement campaign from 9 to 22 of January 2013 is presented in Figure 13. The sampling site is at the center of the pollution-concentration wind rose. The wind direction is toward the center of the rose. The sector length indicates the frequency of wind direction (%) and the colors correspond to different CH_4_ concentrations (ppm). Decomposition color of each wind sector gives the frequency of each concentration range over all measurements.

For example, we can observe that approximately 20% of the measured CH_4_ concentration corresponding to an easterly wind (E), in which ~5% and ~6% of CH_4_ concentrations were between 2.0 to 2.2 ppm and 1.6 to 1.8 ppm, respectively.

This pollution-concentration wind rose indicates that the contribution of local industries from N–WSW sectors to higher CH_4_ concentration is relatively low (except for the NW sector), the higher CH_4_ level (>1.8 ppm) are mainly due to urban emissions from the regions of E–SSE urban areas, which was the dominant CH_4_ source for the Dunkirk area during the present campaign in winter.

## 4. Conclusions

This work focused on the instrumental development, the performance characterization and the field deployment of an optical sensor based on infrared DFB-QCL operating at a wavelength suitable for sensitive and selective quantitative detection and monitoring of CH_4_ in atmospheric environment. The developed DFB-QCL CH_4_ sensor, designed for ease of instrument operation and data acquisition/analysis, is based on a simple multipass direct absorption spectroscopic approach which renders the sensor self-calibrated. It operates at thermoelectrically cooled room-temperature and offers the ability of fast frequency tuning at the kHz scanning rate of spectral data acquisition. In addition, it is mode-hop free frequency tunable over 5 cm^−1^ around 1254 cm^−1^ providing multi-species (CH_4_, HONO, N_2_O, H_2_O) sensing ability, combined with “TeamViewer” remote controlling mode for 24/7 service operation, which truly makes the sensor cost-effective.

The developed sensor was deployed in a campaign of measurements of time series CH_4_ concentration on a site near a suburban traffic road in Dunkirk. An average CH_4_ concentration of (1.77 ± 0.20) ppm was measured during the 14 days’ campaign. The episode of the highest CH_4_ concentration of up to ~3 ppm, observed on 17 January, has been analyzed by combining local meteorological data with regional back trajectory calculations. The higher CH_4_ concentration was mainly attributed to urban pollution transported by air mass through local urban area and the district of Lille, which was confirmed by the pollution wind rose of CH_4_ concentrations in relation to wind sector and by the analysis of air mass back trajectories using the HYSPLIT trajectory model as well as by the analysis of the air quality monitoring data measured at a regional network site in Lille.

The understanding and inventory of CH_4_ sources reflects mixed effects of photochemical reactions, transport patterns and processes, atmospheric dispersion, *etc.* Recent study [31] shows that the CH_4_ emissions from natural gas are 2–3 times larger than predicted by existing inventory methodologies and industry reports. These findings suggest that natural-gas-consuming regions may be larger sources of CH_4_ to the atmosphere than is currently estimated and represent areas of significant resource loss. This is a serious issue that should be urgently addressed with the local authorities and energy supply agencies, in particular in the context of the local installation and operation of a LNG terminal.
